# Parasite and host elemental content and parasite effects on host nutrient excretion and metabolic rate

**DOI:** 10.1002/ece3.3129

**Published:** 2017-06-22

**Authors:** Nicole Chodkowski, Randall J. Bernot

**Affiliations:** ^1^ Department of Biology Ball State University Muncie IN USA

**Keywords:** Ecological stoichiometry, *Elimia livescens*, growth rate hypothesis, metabolism, nutrient excretion, parasites, trematode

## Abstract

Ecological stoichiometry uses the mass balance of elements to predict energy and elemental fluxes across different levels of ecological organization. A specific prediction of ecological stoichiometry is the growth rate hypothesis (GRH), which states that organisms with faster growth or reproductive rates will require higher phosphorus content for nucleic acid and protein synthesis. Although parasites are found ubiquitously throughout ecosystems, little is understood about how they affect nutrient imbalances in ecosystems. We (1) tested the GRH by determining the carbon (C), nitrogen (N), and phosphorus (P) content of parasitic trematodes and their intermediate host, the freshwater snail *Elimia livescens*, and (2) used this framework to determine the trematode effects on host nutrient excretion and metabolism. Snail and parasite tissues were analyzed for elemental content using a CHN analyzer and soluble reactive phosphorus (SRP) methods. Ammonium and SRP assays were used to estimate N and P excretion rates. A respirometer was used to calculate individual snail metabolism. Trematode tissues contained lower C:P and N:P (more P per unit C and N) than the snail tissues. Snail gonadal tissues more closely resembled the elemental content of parasite tissues, although P content was 13% higher in the gonad than the trematode tissues. Despite differences in elemental content, N and P excretion rates of snails were not affected by the presence of parasites. Parasitized snails maintained faster metabolic rates than nonparasitized snails. However, the species of parasite did not affect metabolic rate. Together, this elemental imbalance between parasite and host, and the altered metabolic rate of infected snails may lead to broader parasite effects in stream ecosystems.

## 1 INTRODUCTION

Parasites affect the behavior, morphology, life history, and survival of their hosts (e.g., Bernot, 2003; Lafferty & Kuris, 2009; Mirura, Kuris, Torchin, Hechinger, & Chiba, 2006). Increasingly, evidence that these parasite‐induced trait shifts cascade to community and ecosystem effects is pointing to a more influential and broader ecological role of parasites (Bernot & Lamberti, 2008; Wood & Johnson, 2015). Parasites mechanistically alter their hosts in ways that range from both inconspicuously altering host brain chemistry that results in changes in host behavior to taking over or “snatching” a significant of portion host body tissue while castrating them (Lafferty & Kuris, 2009).

Beyond behavioral and trait shifts, parasites may also affect the nature of the way animals function in the ecosystem, with parasitized individuals expressing a different functional phenotype than nonparasitized individuals (Lafferty & Kuris, 2009). Recent efforts have studied parasite–host interactions in terms of nutrient balance (Bernot, 2013) that can be scaled up to ecosystems (Mischler, Johnson, McKenzie, & Townsend, 2016). For example, snails infected with a trematode parasite recycled nutrients at distinctly different rates than uninfected snails, illustrating a functional difference caused by the parasite (Bernot, 2013). Similarly, parasites have long been studied for their effects on host metabolic rate that represents energy flow within hosts. Together, nutrient cycling and energy flow are the metrics that connect cellular, organismal, and ecosystem levels of organization, making them ideal response variables to measure in ecological parasitology studies.

Ecological stoichiometry (ES) is the study of the balance of energy and elemental fluxes among interacting organisms that provides the framework for predictions at multiple levels of biological organization from the cellular level to the entire ecosystem (Bernot, 2013; Sterner & Elser, 2002). The majority of ES studies have focused on carbon (C), nitrogen (N), and phosphorus (P) as potential limiting nutrients of living things, although other elements such as iron or calcium may also limit organismal fitness (Prater, Wagner, & Frost, 2016). At the cellular and organismal levels, the growth rate hypothesis (GRH) uses the ES framework to predict that organisms invest in phosphorus‐rich ribosomes and rRNA to support the rapid protein synthesis associated with fast growth (Sterner & Elser, 2002). Thus, fast‐growing organisms tend to contain lower tissue N:P ratios compared to slower‐growing or nongrowing organisms. In a similar vein, organisms that divert growth toward rapid reproduction should also use more P, or at least sequester more P in reproductive tissue relative to somatic tissue in preparation for rapid protein synthesis (increased “yield” sensu Elser, Sterner, et al., 2000). While reproductive tissues, such as eggs, of some animals are no different in nutrient content than somatic tissues (Færøvig & Hessen, 2003), rapidly reproducing animals such as hermaphroditic mollusks have eggs with elevated P content (Thompson & Lee, 1985). At a broader ecosystem level, animal stoichiometry affects the ecological functioning of an animal (see reviews Elser et al., 2003; Hessen, Elser, Sterner, & Urabe, 2013). For example, animals excrete N and P at different rates and ratios due to differences in resource content and animal homeostatic elemental needs (Elser & Urabe, 1999; Frost, Evans‐White, Finkel, Jensen, & Matzek, 2005; Persson et al., 2010). Thus, animals with low N:P body composition may retain P and recycle N liberally depending on food nutrient content and animal growth rate (Sterner & Elser, 2002).

Parasites may have different elemental content than their host, thereby leading to either changes in host food consumption to compensate for the parasite or changes in host recycling rates or ratios of limiting nutrients. For instance, elemental changes in *Daphnia* N and P were related to bacteria‐induced castration (Frost, Ebert, & Smith, 2008a,b). Alternatively, body nutrient content of the snail *Physa acuta* was not altered by the trematode *Trichobilharzia physellae,* but infected snails excreted N more rapidly and P less rapidly than uninfected snails (Bernot, 2013). Similarly, N excretion increased in *Stagnicola elodes* snails infected with the trematode *Cotylurus flabelliformis* (Mischler et al., 2016). Therefore, members of the same species may function differently depending on their infection status, which can scale up to ecosystem nutrient flux (Bernot, 2013; Mischler et al., 2016).

Digenetic trematodes are one group of parasites that can be present in relatively large biomasses in aquatic ecosystems (Kuris et al., 2008; Preston, Orlofske, Lambden, & Johnson, 2013). Trematodes (Platyhelminthes: Digenea) have complex life cycles that maintain life stages in mollusk intermediate and vertebrate definitive hosts (Bush, Fernández, Esch, & Seed, 2001). Within the intermediate host, parasites undergo rapid, asexual reproduction and replace or displace significant portions of host biomass (Kuris et al., 2008; Preston et al., 2013), particularly within the gonadal tissues. This “body snatching” (sensu Lafferty & Kuris, 2009) effectively castrates the snail host and reduces host fitness while using the snail body as a vessel for improving their own fitness by reallocating energy for their own growth and reproduction.

Our objectives were to (1) compare the elemental composition (C:N:P) of different tissues of a host to parasite tissue while testing the growth rate hypothesis, and (2) determine the parasite effects on host functioning in the form of nutrient excretion and metabolic rate. We used a freshwater snail *Elimia livescens* (Gastropoda: Pleuroceridae) and common trematode parasites that invade snail tissue as a model host–parasite system. We predicted that as larval trematode rediae and sporocyst stages undergo rapid asexual reproduction, which should require more protein synthesis demands than other life stages of the trematode or other tissues, these stages will contain more P. However, we expect parasite tissues will closely resemble snail gonadal tissues because those tissues are the site of snail reproduction in uninfected individuals. We predicted that parasitized snails would excrete lower C:N:P ratios than nonparasitized snails, as they would retain P for rapid reproduction of the parasite. Parasites may also exert a metabolic demand on the host, leading to faster metabolic rates in parasitized snails. This information will provide better insight into parasite effects on nutrient dynamics and energy flow in stream ecosystems.

## 2 MATERIALS AND METHODS

### 2.1 Study sites and general procedure sequence

*Elimia livescens* (length 12.4–23.2 mm; *n* = 100) snails were collected at three sites along the White River, Muncie, IN (McCulloch Park [MP]: 40°12′4.07″N 85°22′49.28″W; Bureau of Water Quality [BWQ]: 40°11′7.28″N 85°26′17.91″W; South Burlington Ave [SB]: 40°10′2.70″N 85°20′29.57″W), and at a tributary of the White River (Truitt Ditch [TD]: 40°11′14.1″N 85°20′47.3″W) from 22 July 2015 to 13 August 2016. These sites were all in close proximity to each other (within 2 km) and connected via river or stream reach and were not different from each other in physicochemical characteristics (N. Chodkowski, unpublished data). Within 48 hr of collection, snail shell and aperture lengths, wet biomass, and biovolume were measured and then placed into a Qubit Systems respirometer to measure oxygen consumption as an estimate of metabolic rate. Next, snails were placed into individual containers to measure N and P excretion rates and ratios. Finally, each snail was dissected to separate snail foot, snail gonadal tissues, and trematodes for C, N, and P elemental analysis.

### 2.2 Snail metabolic function

Snails were collected and held in aerated river water without food for up to 48 hr before measuring metabolism. Algae were removed from shells before snails were placed into the Qubit Systems respirometer to determine oxygen consumption and standard metabolic rate of uninfected and infected individuals for 30 min. For each snail, the respirometer was flushed for one minute before continuously collecting data for 10 min; this was repeated 3× per snail, and the average slope of the line was calculated between 120 s and 660 s. The volume of oxygen consumed (mg of oxygen/snail biomass kg/hour) was calculated using the following equation:

$${\text{VO}}_{2}=\text{DO slope}\times \left(V_{r}\;-\;V_{a}\right)\;\times \;\left(3600÷m\right)$$

where DO slope is the rate of decrease in dissolved oxygen (mg O_2_ L^−1^ s^−1^) at constant water temperature (20°C), *V*_r_ is the volume of the respirometer chamber (0.215 L), *V*_a_ is the biovolume of the snail, m is the snail biomass (kg).

### 2.3 Nitrogen and phosphorus excretion

To measure ammonium (N) and phosphorus (P) excretion rates, snails were individually housed in freshwater (200 ml; 25 ml river water and 175 ml aged tap water) for three hours (Bernot, 2013). We placed snails in the water mixture to obtain measurable N and P excretion values, while mitigating excess excretion due to stress. After excretion, the water was filtered through Whatman^™^ GF/F 0.7‐μm filters to remove any particulates including possible trematode cercariae. Initial and final N and P concentrations of the water samples (50 ml) were measured using the phenate hypochlorite method for ammonium (Solórzano, 1969) and molybdate blue–ascorbic acid method for soluble reactive phosphorus (APHA 2002). Nutrient excretion rates were calculated from excretion numbers based on snail dry biomass.

### 2.4 Elemental composition of snail and trematode tissues

Snails were dissected and examined for trematode parasites in the gonadal and digestive tissues and surrounding body cavity. Parasite taxa were identified by examining cercariae morphology using light microscopy (dichotomous keys were used from Schell, 1970). Shell length and the presence of external metacercarial cysts formed by trematode *Macravestibulum obtusicaudum* were recorded. The head and foot (collectively referred to as the foot) of the snail were separated from the gonadal and digestive (collectively referred to as the gonad) tissues. For parasitized snails, trematode rediae and/or sporocysts were removed from the infected tissues. Snail and parasite tissues were dried at 60°C for at least 24 hr. Tissue samples were analyzed using the Series II CHNS/O elemental analyzer (PerkinElmer CHN 2400, PerkinElmer Health Sciences Inc., Shelton, Connecticut) to determine carbon (C) and nitrogen (N) content. Phosphorus content was determined by tissue digestion using the molybdate blue–ascorbic acid method after hydrochloric acid digestion (American Public Health Association (APHA), 2002).

### 2.5 Statistical analyses

Preliminary analyses that used the different sites as an explanatory variable in a mixed model revealed no site effect for any of our response variables. Thus, sites were not included in the results presented here. Elemental content (C, N, P), elemental ratios (C:N, C:P, N:P), N and P excretion rates, and metabolic rates were checked for normality using a Shapiro–Wilk normality test. Wilcoxon signed‐rank test for nonparametric data and a Student's *t* test for parametric data were used to determine differences between snail foot and gonadal tissues. Analysis of variance (ANOVA) was used to determine whether snail foot, snail gonad, and trematode tissues differed in elemental content and ratios. Pairwise differences between tissue types were determined with Tukey's post hoc comparisons. Differences in nutrient excretion rates were determined for N and P using analysis of covariance (ANCOVA) to determine whether trematodes influenced nutrient excretion while accounting for snail size. Analysis of covariance was also used to determine whether parasitized snails maintained faster metabolic rates than nonparasitized snails while accounting for differences in snail size. Regression analysis was used to determine whether N and P excretion rates and metabolic rate depended on parasite load in parasitized snails where parasite load was calculated as the percent of total dry biomass that was parasite tissue. All analyses were conducted in R (R Development Core Team, 2008, ver. 2.14.1).

## 3 RESULTS

A total of 100 *Elimia livescens* snails were examined for elemental content, nutrient excretion, and metabolic rates. Of these, 46 snails contained trematodes: *Macrovestibulum obtusicaudum* (Pronocephalidae; *n* = 18), *Protometra macrostoma* (Azygiidae; *n* = 13), *Acanthatrium oregonense* (Lecithodendriidae; *n* = 6), unknown monostome cercariae (*n* = 3), and *Azygia* sp. (Azygiidae; *n* = 2). Some snails were coinfected with either *M. obtusicaudum* and *A*. *oregonense* (*n* = 3) or *M. obtusicaudum* and *P. macrostoma* (*n* = 1). Larger snails were more likely to have parasites than smaller snails (*t* = −4.67, *df* = 78.38, *p <* .01).

### 3.1 Elemental composition of snail and parasite tissues

**Figure 1:**
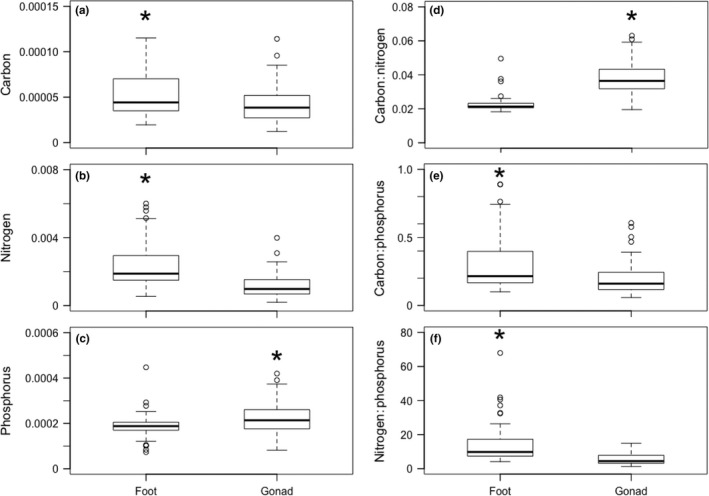
Boxplots of *Elimia livescens* foot and gonadal tissue molar C (a), molar N (b), molar P (c), molar C:N (d), molar C:P (e), and molar N:P (f) from nonparasitized (*n* = 54) snails collected from three sites in the White River, Indiana, during July and August 2015. Boxplots with the asterisk indicate higher elemental content

The elemental compositions differed between foot and gonadal tissues of nonparasitized snails. The C (*W =* 1832.5, *p <* .02, Figure 1a) and N (*W =* 2,424, *p <* .01, Figure 1b) contents in nonparasitized snail foot tissues were greater than in snail gonadal tissues. However, the gonadal tissues contained more P than the foot (*W =* 989, *p <* .01, Figure 1c). The C:N (*W =* 166, *p <* .01, Figure 1d) and N:P (*W =* 2,470, *p <* .01, Figure 1f) ratios were greater in nonparasitized snails than parasitized snails, but C:P (*W =* 1990, *p <* .01, Figure 1e) was greater in parasitized snails than nonparasitized snails.

**Figure 2:**
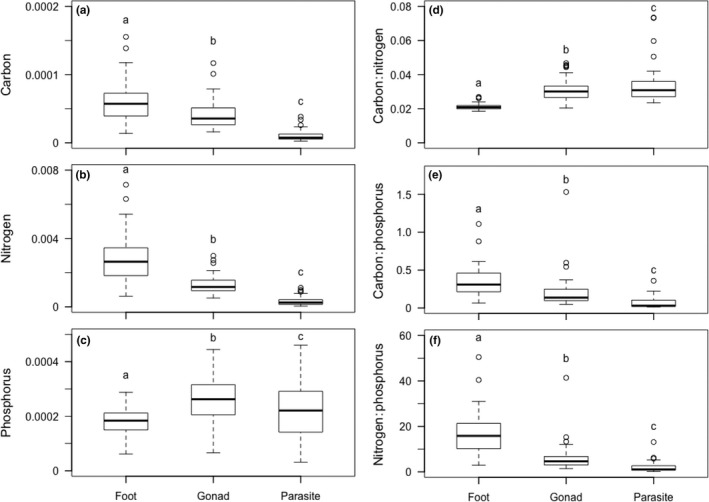
Boxplots of *Elimia livescens* foot, gonadal, and trematode tissue molar C (a), molar N (b), molar P (c), molar C:N (d), molar C:P (e), and molar N:P (f) from parasitized snails (*n* = 46) collected from three sites in the White River, Indiana, during July and August 2015. Boxplots with the same letter are not different

The elemental content of parasitized snail foot and snail gonadal tissues differed from trematode tissues. The C (χ^2^ (2)* =* 88.54, *df* = 2, *p <* .01, Figure 2a), N (χ^2^ (2)* =* 102.61, *df* = 2, *p <* .01, Figure 2b), P (χ^2^ (2)* =* 21.69 *df* = 2, *p <* .01, Figure 2c), C:N (χ^2^ (2)* =* 84.02, *df* = 2, *p <* .01, Figure 2d), C:P (χ^2^ (2) = 76.88, *df* = 2, *p <* .01, Figure 2e), and N:P (χ^2^ (2)* =* 93.89, *df* = 2, *p <* .01, Figure 2f) differed among foot, gonad, and trematode tissues. Snail foot tissues contained more C and N per unit biomass than the snail gonad (*p <* .01) or trematode (*p <* .01) tissues. Gonadal tissues also contained more C (*p <* .01) and N (*p <* .01) than trematode tissues. Phosphorous content was also 44% greater in the gonad than the foot (*p <* .01) and 13% greater in trematode tissues (*p <* .01). The C:N was greater in gonad and trematode tissues than the foot (*p <* .01), but there was no difference in C:N between the gonad and trematode tissues (*p =* 0.47). Trematode tissues also had lower C:P and N:P than the snail foot (*p <* .01) and gonad (*p <* .01) tissues driven by the relatively high P content in the trematode tissues. Because the gonad tissues more closely resembled that of trematode tissues, the gonadal tissues maintained lower C:P (*p <* .01) and N:P (*p <* .01) than foot tissues.

### 3.2 Nutrient excretion rates of parasitized and nonparasitized snails

**Figure 3:**
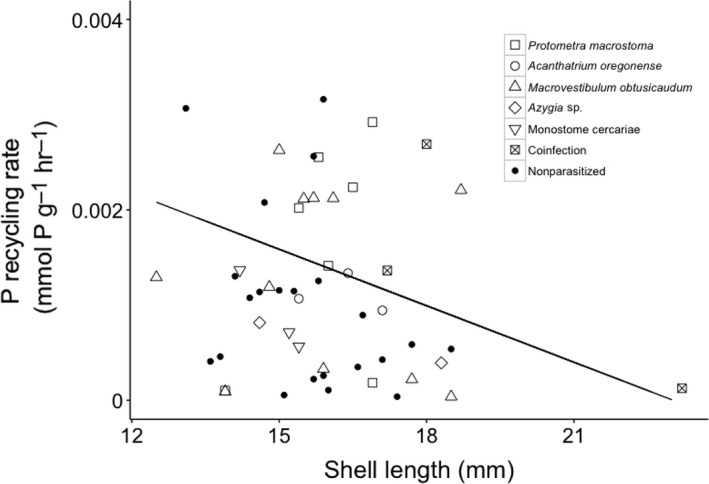
Scatterplot of phosphorus excretion rates vs. shell length of *Elimia livescens* snails. Line represents a linear relationship between *E. livescens* P excretion rates and shell length (P excretion = −0.0002(shell length) + 0.0042; *R* ^2^ = .07345)

Nitrogen excretion rates were measured from 79 snails, and P excretion rates were estimated from 53 snails. Nitrogen (*F*_1,75_ = −0.13, *p =* .19) and P (*F*_1,50_ = 0.48, *p =* .49, Figure 3) excretion rates did not differ between parasitized and nonparasitized snails. However, P excretion rates were faster in snails with shorter shell lengths (*F*_1,50_ = 4.47, *p =* .04, Figure 3). There was no relationship between shell size and N excretion rates (*F*_1,75_ = 0.29, *p =* .77). Phosphorus and N excretion rates of snails did not differ when parasitized by different trematode taxa (*F*_5,42_ = 1.55, *p* = .20). Parasite load did not affect N (*F*_1,27_ = 0.21, *p =* .65) or P (*F*_1,20_ = 0.18, *p =* .68) excretion rates.

### 3.3 Metabolic rates of parasitized and nonparasitized snails

**Figure 4:**
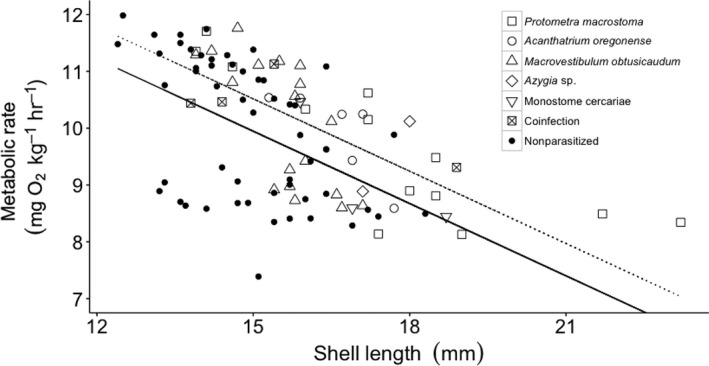
Scatterplot of metabolic rate as the volume of oxygen consumed (VO_2_) vs. shell length of *Elimia livescens* snails. Lines represent linear relationships in metabolic rate of *E. livescens* with (metabolic rate = −0.1768(shell length) + 6.0944, *R* ^2^ = .40072; dotted black line) and without (metabolic rate = −0.1829(shell length) + 5.9846, *R* ^2^ = .22463; solid black line) trematode infection

Metabolic rates were measured from 100 snails. Parasitized snails exhibited faster metabolic rates than nonparasitized snails (ANCOVA: *F*_1,97_ = 7.17, *p* < .01, Figure 4). Metabolic rates were also negatively related to shell length (ANCOVA: *F*_1,97_, *p* < .01, Figure 4). However, the identity of the parasite did not affect metabolic rates of snails (*F*_5,34_ *=* 1.91, *p =* .12, Figure 4). Parasite load did not affect snail metabolic rate (*F*_1,34_ = 0.18, *p =* .84).

## 4 DISCUSSION

The growth rate hypothesis of ES predicts that relatively fast‐growing tissue harbors more P per unit N due to rapid cell division. Our results with *Elimia* and its trematode parasites support the GRH in that snail gonadal tissues contained more P and closely resembled the parasite tissues, which also contained greater P content. Functionally, parasitized snails did not excrete N or P at different rates than nonparasitized snails, which was not consistent with a previous study in the same system but with different parasites and hosts (Bernot, 2013). However, parasitized snails exhibited faster metabolic rates than their nonparasitized counterparts, consistent with our prediction.

Our study found elevated C content in infected snail tissues, but both infected and uninfected individuals maintained similar N and P elemental composition. In *Physella* snails, trematode infection increased P content in infected individuals (Narr & Krist, 2015), but elemental content remained stable among all tissue types of infected and uninfected *Physa acuta* (Bernot, 2013). Although infection status alone did not change host tissues, there were discernable elemental differences between snail host and parasitic trematode tissues; trematodes maintained less C and N, but had more P per unit C and per unit N relative to the host tissues. These findings aligned with the elevated P content in trematodes of *P. acuta* snails (Bernot, 2013). The trematode tissues compositionally resembled the gonadal tissue of the host, which is the site of trematode development and reproduction. Similarly, the δ^15^N isotopic signatures of the hepatopancreas (the site of trematode growth and replication) in *Lymnaea stagnalis* were higher than the hepatopancreas from uninfected individuals, and more closely resembled the enriched δ^15^N found in the parasite tissues (Doi et al., 2008). In contrast, parasite δ^15^N tissues were lower than that of the bivalve cockle (*Cerastoderma edule*) and gastropod dogwhelk (*Nassarius reticulatus*) hosts (Dubois et al., 2009), which is similar to the relatively low N content of parasites to snail host found in our study.

Aquatic systems are generally P‐limited (Elser, Fagan, et al., 2000), which may hinder organismal growth and fecundity. Phosphorus content and RNA are related to growth rates at cellular, developmental, organismal, and ecological scales (Elser et al., 2003). Therefore, P‐limitations can negatively affect host organisms and parasites, alike. For instance, freshwater snails, *Radix ovate,* fed low‐P diets grew slower than nonnutrient‐depleted snails (Fink & Von Elert, 2006). Likewise, growth was hindered for mussels living in nutrient‐poor waters (Strayer, 2014). Infected *Physella* snails fed high‐P diets released more cercariae than infected snails fed low‐P diets (Narr & Krist, 2015). However, our system is not N‐ or P‐limited, nor was there differences in N or P excretion rates between parasitized and nonparasitized *Elimia*. Thus, parasitism in this system is unlikely to ameliorate or exacerbate nutrient limitation.

In other studies, parasites promoted intraspecific differences in nutrient excretion and biogeochemical cycling through consumer‐driven nutrient recycling (CDNR) (Preston, Mischler, Townsend, & Johnson, 2016). *Physa acuta* snails collected from the same river in Muncie, IN, excreted N and P faster than the *E. livescens* in this study (Bernot, 2013). Contrary to our results, infected snail hosts in other studies increased N excretion and reduced P excretion (Bernot, 2013; Mischler et al., 2016). For example, uninfected *P. acuta* excreted N at a mean rate of 2.24 mmol N g^−1^ hr^−1^, which was slower than the 4.66 mmol N g^−1^ hr^−1^ excreted by infected snails of the same population (Bernot, 2013). Both these rates, however, are faster than then 0.001 mmol N g^−1^ hr^−1^ from uninfected and 0.00077 mmol N g^−1^ hr^−1^ from infected *E. livescens* snails in our study collected from the same habitat. Against our predictions, P excretion rates were not different between infected and uninfected *E. livescens* (both means = 1.3 × 10^−3^ mmol P g^−1^ hr^−1^), but this rate was slower than mean uninfected (0.17 mmol P g^−1^ hr^−1^) and infected (0.22 mmol P g^−1^ hr^−1^) *P. acuta* P excretion rates (Bernot, 2013). Unlike our study, snail shell length did not affect P excretion rates (Bernot, 2013).

Interestingly, under high N and P nutrient conditions, fish generally excrete N roughly an order of magnitude faster (0.012 mmol N g^−1^ hr^−1^) than *E. livescens* (Vanni, Flecker, Hood, & Headworth, 2002). However, P excretion rates were an order of magnitude lower (1.87 × 10^−4^ mmol P g^−1^ hr^−1^) (McIntyre et al., 2008), potentially attributed to the greater P content required for bone in vertebrates (Sterner & Elser, 2002). Moreover, the aggregate nutrient excretion rates can be dependent on organismal densities within an area, thereby creating biogeochemical hot spots (McIntyre et al., 2008). Differences in life spans, ecological function and distribution, and body elemental composition,between snail species, could help explain the observed differences in excretion rates.

Metabolism governs organismal cellular work, which ultimately scales to their function in ecosystems. The metabolic theory of ecology (MTE), in conjunction with ES, could be used to predict changes in nutrient storage, flux, and turnover in ecosystems (Allen & Gillooly, 2009; Hechinger, 2013). *Elimia livescens* are operculate freshwater snails and maintain slower (>1 order of magnitude slower) metabolic rates than pulmonate snails (Von Brand, Nolan, & Mann, 1948). For instance, the average metabolic rate for our population of *E. livescens* snails was 37,000 mg O_2_ kg^−1^ hr^−1^, while metabolic rates of air‐breathing species *Biomphalaria glabrata* (Family: Planorbidae) and *L. stagnalis* (Family: Lymnaeoidea) (at 25°C) were roughly 175,000 mg O_2_ kg^−1^ hr^−1^ (Von Brand et al., 1948) and 200,000 mg O_2_ kg^−1^ hr^−1^ (Duerr, 1967), respectively. These differences could be due to structural or functional differences in obtaining oxygen between these phylogenetically distinct groups of snails. In our study, larger snails had slower metabolic rates, which is typical for snail intraspecific comparisons (Speakman, 2005; Von Bertalanffy, 1957; Von Brand et al., 1948). Similarly, isopods infected with acanthocephalan parasites increased foraging and respiration (Lettini & Sukhdeo, 2010). Parasites, therefore, may alter the metabolic function and energy allocations of their hosts, which can both be influenced by nutrient availability and affect energy flow through ecosystems.

Larger *E. livescens* were more likely to be infected than smaller snails, which is consistent with other *E. livescens* populations (Huehner, 1987). These infected snails could be larger as a consequence of gigantism, a phenomenon that results in rapid growth of infected individuals after castrator parasites “snatch” their hosts’ body. Gigantism energetically favors parasite fecundity, but it negatively affects survival and reproduction of the host (Ebert, Carius, Little, & Decaestecker, 2004; Hall, Becker, & Cáceres, 2007). The larger body size of the host may serve as the extended phenotype of the parasite to allow for rapid growth of trematodes compared to the uninfected snails (Hechinger, 2010; Lafferty & Kuris, 2009).

Ecological stoichiometry is a useful framework for understanding trophic dynamics and nutrient recycling in aquatic ecosystems (Elser & Urabe, 1999; Hessen et al., 2013). Determining the elemental content in parasite and host tissues will help us determine elemental imbalances between uninfected and infected hosts, and how these imbalances may influence nutrient cycling at larger scales. Aquatic systems may be more N‐limited than previous thought (Elser, Fagan, et al., 2000) and may limit the ability of organisms to build proteins (Elser et al., 2003) and maintain metabolism (Anderson, Hessen, Elser, & Urabe, 2005). Overall, trematode tissues retained lower C:P and N:P than the *E. livescens* intermediate snail host tissues, which is likely attributed to the proportionally large P required for the trematodes to undergo rapid, asexual reproduction. Although infection status had no effect on the nutrient excretion rates of the snail hosts, parasitized snails maintained faster metabolic rates than nonparasitized individuals, suggesting differential energy provisioning. The elemental imbalances between parasite and host tissues, and the altered metabolic function of infected snail hosts, suggest parasites may affect energy dynamics in lotic ecosystems.

## AUTHORS’ CONTRIBUTIONS

Both N. Chodkowski and R.J. Bernot generated the ideas and designed the methodology; N. Chodkowski collected the data and headed the writing of the manuscript; N. Chodkowski, with the help of R.J. Bernot, analyzed the data. Both authors contributed critically to the drafts and gave final approval for publication.

## DATA ACCESSIBILITY

These data and R script used to run the analyses can be accessed through the Dryad Digital Repository.

## CONFLICT OF INTEREST

None declared.
